# Fatty liver index and development of cardiovascular disease in Koreans without pre-existing myocardial infarction and ischemic stroke: a large population-based study

**DOI:** 10.1186/s12933-020-01025-4

**Published:** 2020-05-02

**Authors:** Jun Hyung Kim, Jin Sil Moon, Seok Joon Byun, Jun Hyeok Lee, Dae Ryong Kang, Ki Chul Sung, Jang Young Kim, Ji Hye Huh

**Affiliations:** 1 Division of Cardiology, Department of Internal Medicine, Yonsei University Wonju College of Medicine, Wonju, 220-701 Republic of Korea; 2 Department of Biostatistics, Yonsei University Wonju College of Medicine, Wonju, 220-701 Republic of Korea; 3 Department of Precision Medicine & Biostatistics, Yonsei University Wonju College of Medicine, Wonju, 220-701 Republic of Korea; 4 Division of Cardiology, Department of Internal Medicine, Kangbuk Samsung Hospital, Sungkyunkwan University School of Medicine, Seoul, Republic of Korea; 5 Division of Endocrinology and Metabolism, Department of Internal Medicine, Hallym University Sacred Heart Hospital, Anyang, 14068 Republic of Korea

**Keywords:** Cardiovascular disease, Mortality, Non-alcoholic fatty liver disease, Fatty liver index

## Abstract

**Background:**

Despite the known association between non-alcoholic fatty liver disease (NAFLD) and cardiovascular disease (CVD), whether NAFLD predicts future CVD events, especially CVD mortality, remains uncertain. We evaluated the relationship between fatty liver index (FLI), a validated marker of NAFLD, and risk of major adverse cardiac events (MACEs) in a large population-based study.

**Methods:**

We identified 3011,588 subjects in the Korean National Health Insurance System cohort without a history of CVD who underwent health examinations from 2009 to 2011. The primary endpoint was a composite of cardiovascular deaths, non-fatal myocardial infarction (MI), and ischemic stroke. A Cox proportional hazards regression analysis was performed to assess association between the FLI and the primary endpoint.

**Results:**

During the median follow-up period of 6 years, there were 46,010 cases of MACEs (7148 cases of cardiovascular death, 16,574 of non-fatal MI, and 22,288 of ischemic stroke). There was a linear association between higher FLI values and higher incidence of the primary endpoint. In the multivariable models adjusted for factors, such as body weight and cholesterol levels, the hazard ratio for the primary endpoint comparing the highest vs. lowest quartiles of the FLI was 1.99 (95% confidence interval [CIs], 1.91–2.07). The corresponding hazard ratios (95% CIs) for cardiovascular death, non-fetal MI, and ischemic stroke were 1.98 (1.9–2.06), 2.16 (2.01–2.31), and 2.01 (1.90–2.13), respectively (p < 0.001). The results were similar when we performed stratified analyses by age, sex, use of dyslipidemia medication, obesity, diabetes, and hypertension.

**Conclusions:**

Our findings indicate that the FLI, which is a surrogate marker of NAFLD, has prognostic value for detecting individuals at higher risk for cardiovascular events.

## Background

Non-alcoholic fatty liver disease (NAFLD) is characterized by the accumulation of fat in the liver attributable to insulin resistance, in the absence of significant alcohol use. NAFLD is currently the most common cause of chronic liver disease globally, and its reported prevalence in the adult population is 20–30%; however, the prevalence can increases up to 70–90% in obese or diabetic patients [1, 2]. NAFLD was previously considered an intra-hepatic phenotype of metabolic syndrome; however, it has since been revealed that NAFLD itself is an independent risk factor for various chronic diseases such as cardiovascular disease (CVD) [3], hypertension [4], diabetes [5, 6], and chronic kidney disease [7]. Additionally, because CVD is the most common cause of death among NAFLD patients, studies of the relationship between NAFLD and CVD and underlying mechanisms have been actively conducted [8].

Because NAFLD has a variable prognosis, it is clinically important to identify subjects with NAFLD. The gold standard for diagnosing NAFLD is liver biopsy. However, liver biopsy is not only difficult but also unnecessary for all patients with NAFLD because of the risk of complications due to its invasive nature, potential for sampling error, and high cost [9]. Therefore, some non-invasive, non-imaging approaches have been studied and applied in the general population to diagnose fatty liver, including the fatty liver index (FLI), SteatoTest, and NAFLD liver fat score [10]. The FLI is a surrogate marker of hepatic steatosis that has been extensively validated in a large group of subjects [11]. Furthermore, several recent studies have demonstrated that as the FLI values increases, the degree of hepatic steatosis worsens [12, 13]. Currently, the FLI is being used in epidemiological studies and for screening the general population as an alternative to ultrasonography.

Regarding the known close association between NAFLD and CVD, several longitudinal studies have shown that steatosis, as assessed by the FLI, occurs before early carotid atherosclerosis and its progression [14]. Pais et al. demonstrated that the FLI effectively predicts intermediate and high Framingham scores [15]. Despite the known usefulness of the FLI as a surrogate marker of NAFLD, there has been no study on the occurrence of CVD using large datasets consisting of more than 1 million people. Furthermore, whether NAFLD is directly associated with the occurrence of mortality caused by CVD is controversial. We conducted a large population cohort study using data from the Korean National Health Insurance Service (NHIS) to extensively investigate the contribution of hepatic steatosis to the risk of CVD-related adverse events, including cardiovascular (CV) deaths.

We studied the prospective association of the FLI with the risk of incident non-fatal myocardial infarction (MI), ischemic stroke, and CV death, as well as the predictive value of the FLI to identify individuals who will develop incident CVD events. The analyses were stratified by age, sex, statin use, and presence or absence of obesity, diabetes, and hypertension. We hypothesized that the FLI would be a predictor of progression to incident CVD in a large population-based cohort.

## Methods

### Study participants

In our cohort study, we used data from the NHIS, which is a government program that was implemented in 2000 and includes data regarding approximately 98% of the Korean population. All clinics, hospitals, and pharmacies in Korea are required to participate in the NHIS, and they are reimbursed for their services through the NHIS after filing claims electronically. Those who are older than 40 years and enrolled in the NHIS are eligible to undergo regular health screenings at least once every 2 years. During this study, the target population was adult men and women older than 40 years who underwent two or more health screenings from 2009 to 2011. The exclusion criteria were as follows: diagnosis of CVD (MI or ischemic stroke) from 2002 to 2009; those for whom we could not calculate the FLI because of missing values; heavy consumption of alcohol (≥ 2 days per week, and more than seven units of alcohol for men and five units for women per day); use of drugs known to cause fatty liver; diagnosis of hepatitis B or hepatitis C. A total of 3014,643 subjects were included in the study. A flow chart of subject selection is depicted in Additional file 1. This study was approved by the Institutional Review Board of Yonsei University Wonju College of Medicine, Republic of Korea (no. CR318352). Anonymous and de-identified data were used for the analysis; therefore, informed consent was not obtained.

### Measurements

Healthcare institutions are designated for screening according to the Framework Act on Health Examinations and must meet the standards for employees, facilities, and equipment [16]. To minimize errors in measurements, the average values of all laboratory test data from 2009 to 2011 were used. Values outside the extreme outlier were treated as missing values. Height, body weight, and waist circumference were measured, and body mass index (BMI) was calculated as the subject’s weight in kilograms divided by the subject’s height in square meters. Blood samples were obtained after an overnight fast for serum glucose and cholesterol level measurements.

### Definition of CV events

We enrolled individuals who underwent two or more health screenings between 2009 and 2011 and who had undergone evaluation of the primary endpoint during the follow-up period from 2014 to 2017. To minimize the influence of possible “reverse causation” (illnesses causing a low FLI), we excluded subjects with CV events that occurred within 3 years after baseline measurements. The primary endpoint was CV events, which was a composite of newly developed CV deaths, MI, and ischemic stroke during the follow-up period. The diagnosis was based on the International Statistical Classification of Diseases and Related Health Problems, 10th revision (ICD-10) codes. MI was determined based on either the recording of ICD-10 code I21 or I22 during hospitalization for ≥ 4 days or the recording of these codes at least twice. Ischemic stroke was determined based on the recording of the ICD-10 code I63 or I64 during hospitalization for ≥ 4 days with claims for brain magnetic resonance imaging or brain computerized tomography [7]. Follow-up evaluations of CV death were based on nationwide death certificate data from the Korea National Statistical Office. Subjects were considered to have completed the study on the date of their CV events or at the end of the follow-up period, whichever came first.

### Calculation of the FLI

According to a previously published report by Bedogni et al. the formula for the FLI is as follows [11]: FLI = [e^0.953^ × log_e_ (triglycerides) + 0.139 × BMI + 0.718 × log_e_ (γ-glutamyltransferase) +0.053 × waist circumference–15.745)]/[1 + e^0.953^ × log_e_ (triglycerides) + 0.139 × BMI + 0.718 × log_e_ (γ-glutamyltransferase) + 0.053 × waist circumference–15.745] × 100; triglyceride levels are presented as mmol/l, γ-glutamyltransferase levels are presented as U/l, and waist circumference measurements are presented as cm. Score ranges from 0 to 100. The values used in the FLI formula were calculated as the mean value of the data measured during the health screenings from 2009 to 2011. Glucocorticoid, tamoxifen, and tetracycline are known to cause fatty liver; hence, we excluded subjects who had any history of using these drugs.

### Statistical analysis

Statistical analysis was performed using SAS 9.4 (SAS Institute Inc., Cary, NC, USA) and R 3.1.0 (R Foundation for Statistical Computing, Vienna, Austria). For each group, the mean and standard deviation are presented for the continuous variables and the frequency and percentages were presented for the categorical variables. Participants were classified into four groups according to the FLI quartiles. To compare each group, we performed two-sample t-test, one-way analyses of variance (ANOVA), and Chi square test, as appropriate. The incidence rate of primary outcomes was calculated by dividing the number of incident cases by the total follow-up duration (person-years). Hazard ratios (HRs) and 95% confidence intervals (95% CIs) for CV death, MI, and stroke were analyzed using the multivariate Cox proportional hazards model for the FLI quartile or decile groups. The multivariable-adjusted proportional hazards models were as follows: model 1 was adjusted for demographic factors such as age and sex; model 2 was adjusted for adjusted for all factors in model 1 and socio-economic factors such as current smoking, regular exercise, and income; and model 3 was adjusted for all factors in models 1 and 2 and further adjusted for clinical factors such as body weight, total cholesterol, presence of diabetes mellitus, presence of hypertension, and use of medication for dyslipidemia. A test for trend was calculated across FLI quartile groups treating the categories as an ordinal variable. Additionally, to reduce the impact of competing risk bias on the result, we performed competing risk model analysis to assess the risk of CV mortality, with death caused by non-CVD considered as a competing event, using subdistribution hazard model by Fine-Gray [17]. The potential modification effects caused by age, sex, obesity, diabetes mellitus, hypertension, and use of lipid-lowering agents were evaluated through a stratified analysis and interaction testing using a likelihood ratio test. In subgroup analyses, the HR (95% CI) of the highest quartile (Q4) was compared with those of the lower three quartiles (Q1–3) as a reference. Results with p-value < 0.05 were defined as statistical significant. The risk was expressed as the 95% CIs.

## Results

### Baseline characteristics

A total of 3011,588 subjects were analyzed in this study. Participants were classified according to the FLI quartiles, and the baseline characteristics are presented in Table 1. The cutoff values for the quartile groups were 8.49, 18.67, and 38.08, and the numbers of subjects in Q1, Q2, Q3, and Q4 were 753,155, 753,007, 752,868, and 752,528, respectively. A total of 1,290,580 (42.9%) subjects were male, and the proportion of males increased with increasing FLI quartiles: Q1 (18.2%, n = 137,010), Q2 (35.3%, n = 265,501), Q3 (50.5%, n = 380,022), and Q4 (67.5%, n = 507,975). At baseline, the mean age was 51.86 ± 8.20 years. The mean age and BMI were higher in higher FLI groups; however, the mean age of subjects in Q4 was slightly lower than that of subjects in Q3. As expected, systolic blood pressure, diastolic blood pressure, fasting glucose, and cholesterol levels were elevated in higher FLI groups. The proportion of subjects who performed regular exercise showed no significant difference between the FLI quartile groups. Regarding the smoking status, the proportion of current smokers was higher in Q4 than in Q2 and Q3; however, this proportion was also high in Q1. The higher the FLI, the greater was the proportion of subjects with hypertension, diabetes, or dyslipidemia.

**Table 1:** Baseline characteristics of subjects according to the fatty liver index (FLI) quartiles

	Total	FLI (Q1)	FLI (Q2)	FLI (Q3)	FLI (Q4)
N	3,011,588	753,155	753,007	752,868	752,528
FLI cutoff value		≤ 8.49	8.5–18.67	18.68–37.08	≥ 37.09
Age (years)	51.86 ± 8.20	49.09 ± 7.38	52.20 ± 8.02	53.35 ± 8.13	52.81 ± 8.15
Body mass index (kg/m^2^)	23.82 ± 2.91	21.05 ± 1.74	23.03 ± 1.75	24.51 ± 1.92	26.69 ± 2.64
Sex (male)	1,290,580 (42.9%)	137,010 (18.3%)	265,501 (35.3%)	380,022 (50.5%)	507,975 (67.5%)
Systolic blood pressure	122.26 ± 12.63	116.00 ± 11.76	121.00 ± 11.99	124.33 ± 11.88	127.73 ± 11.82
Diastolic blood pressure	76.20 ± 8.27	72.36 ± 7.86	75.30 ± 7.86	77.37 ± 7.75	79.77 ± 7.77
Fasting glucose	98.10 ± 20.19	91.77 ± 12.96	95.65 ± 16.81	99.37 ± 20.14	105.63 ± 25.88
Total cholesterol	199.61 ± 33.64	189.32 ± 30.56	198.30 ± 31.92	202.87 ± 33.59	207.94 ± 35.46
Triglyceride	111 (80.67, 156)	72.67 (58.67, 90)	77.5 (80, 124)	127 (101,161)	177 (135.5, 236.5)
HDL cholesterol	55.56 ± 20.92	61.72 ± 17.75	56.80 ± 18.79	53.38 ± 21.00	50.32 ± 23.87
LDL cholesterol	119.49 ± 31.68	112.75 ± 28.76	121.28 ± 30.31	123.59 ± 31.56	120.36 ± 34.76
Estimated GFR (mL/min/1.73 m^2^)	81.28 ± 23.28	74.53 ± 19.87	78.34 ± 21.46	82.08 ± 22.66	90.16 ± 25.80
Income (lower 25%)	843,511 (28%)	239,447 (31.8%)	217,221 (28.9%)	198,702 (26.4%)	188,141 (25.0%)
Current smoker	553,254 (18.4%)	64,933 (8.6%)	109,309 (14.5%)	153,046 (20.3%)	225,966 (30.0%)
Regular exercise (%)	1,668,750 (55.4%)	403,498 (53.6%)	420,508 (55.8%)	424,181 (56.3%)	420,563 (55.9%)
Hypertension (%)	1,086,672 (36.1%)	150,088 (19.9%)	240,205 (31.9%)	311,619 (41.4%)	384,760 (51.1%)
Diabetes mellitus (%)	236,874 (7.9%)	17,827 (2.4%)	40,090 (5.3%)	67,422 (9.0%)	111,535 (14.8%)
Use of medication for dyslipidemia (%)	89,704 (3.0%)	8,293 (1.1%)	18,668 (2.5%)	27,105 (3.6%)	35,637 (4.7%)

Data are expressed as the mean ± SD, median (25–75%), or n (%)

*FLI* fatty liver index, *HDL* high-density lipoprotein, *LDL* low-density lipoprotein

*p-values for the trend were < 0.0001 for all variables except regular exercise

### FLI and primary endpoints

**Fig. 1:**
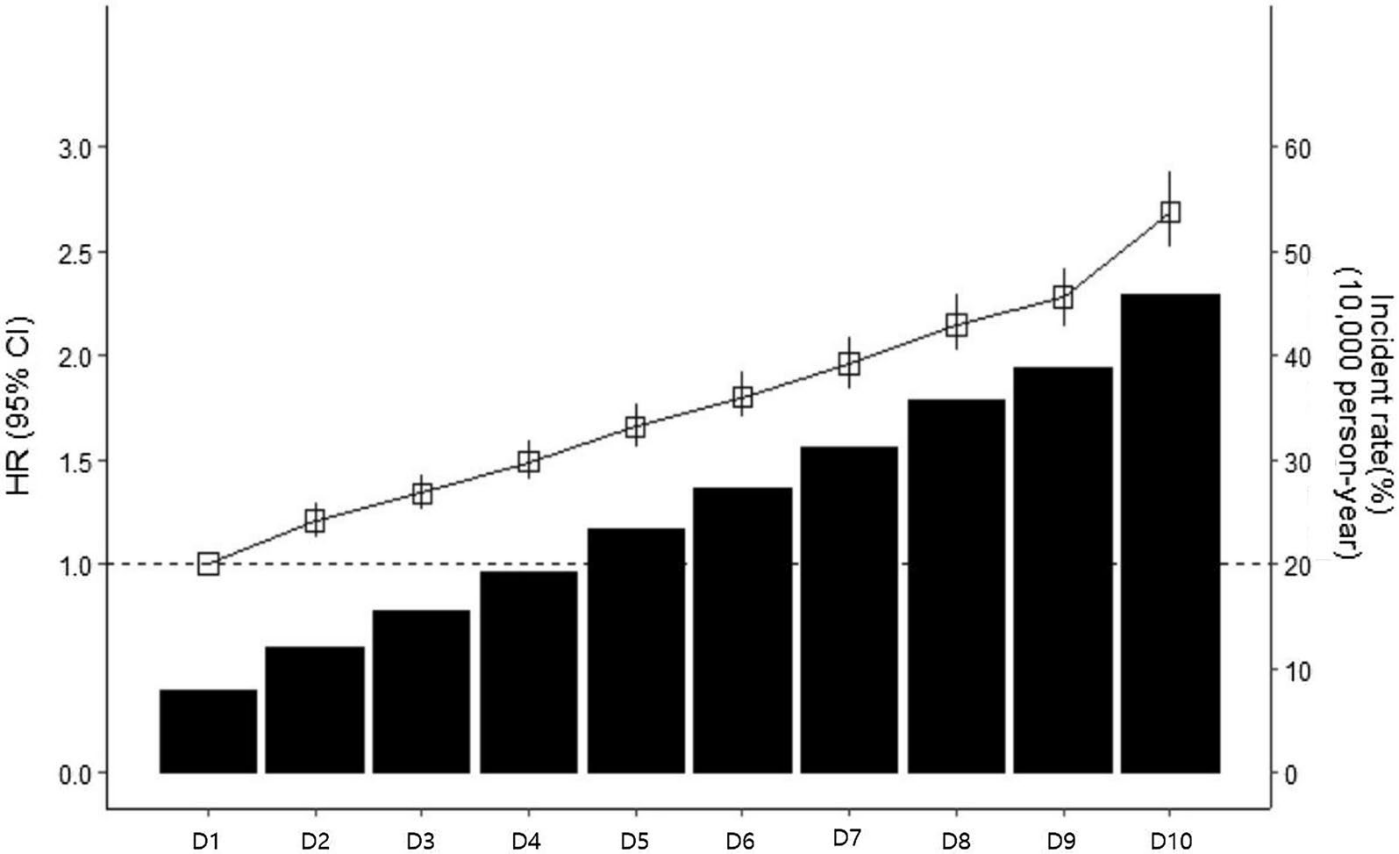
Incidence rates, hazard ratios, and 95% confidence intervals of the primary endpoint (cardiovascular disease mortality, myocardial infarction, and stroke) according to the deciles of the FLI. *FLI* fatty liver index, *HR* hazard ratios, *CI* confidence intervals, *CV* cardiovascular. *Adjusted for age, sex, current smoking, regular exercise, income, body weight, total cholesterol, hypertension, diabetes, and use of medication for dyslipidemia

During the median follow-up of 6 years, there were 46,010 cases of adverse CV events (7,148 CV deaths, 16,574 non-fatal MIs, and 22,228 ischemic strokes) (Table 2). An incrementally higher risk of CV eventd was observed with higher FLI quartiles when compared with Q1 in all models. After adjustment for age, sex, current smoking, regular exercise, income, body weight, total cholesterol, hypertension, diabetes, and medication for dyslipidemia, the relationship between the FLI and adverse CV events still remained significant [HR (95% CI): Q1, reference; Q2, 1.31 (1.27–1.36); Q3, 1.61 (1.55–1.66); Q4, 1.99 (1.91–2.07)]. To determine the linear trends of the risk, we investigated the HRs of primary endpoints according to the FLI decile groups, with the first decile serving as the reference category. The multivariable-adjusted HRs of primary endpoints increased continuously and linearly, and statistical significance was observed from the second decile (D2) of the FLI group (Fig. 1). When this association was stratified by the type of CV event, higher FLI quartiles had a significantly increased risk of non-fatal MI, non-fatal ischemic stroke, and CV deaths. Regarding CV deaths, similar pattern was observed in the competing risk model analysis (Additional file 2). Analyses based on decile groups also demonstrated a linearly increasing risk of all types of CV outcomes in higher FLI decile groups when compared with the lowest decile group (Additional file 3). The risks of MI and stroke significantly increased from the D2 of the FLI group, and the risk of CV mortality significantly increased from the fifth decile (D5) of the FLI group.

**Table 2:** Risk of cardiovascular events (non-fatal myocardial infarction, ischemic stroke, and cardiovascular mortality) according to baseline fatty liver index quartiles

	Events	Incident rate (10,000 person-year)	Unadjusted model HR(95% CI)	Adjusted model HR (95% CI)		
				Model 1	Model 2	Model 3
Total (primary endpoint)						
FLI (Q1)	4870	10.80	Ref	Ref	Ref	Ref
FLI (Q2)	9116	20.27	1.87 (1.81–1.94)	1.35 (1.31–1.40)	1.35 (1.30–1.40)	1.31 (1.27–1.36)
FLI (Q3)	13,535	30.16	2.78 (2.69–2.87)	1.72 (1.66–1.78)	1.71 (1.66–1.77)	1.61 (1.55–1.66)
FLI (Q4)	18,489	41.32	3.80 (3.69–3.93)	2.28 (2.21–2.35)	2.23 (2.16–2.30)	1.99 (1.91–2.07)
p for trend			< 0.0001	< 0.0001	< 0.0001	< 0.0001
Per one SD increase in FLI	46,010	25.60	1.45 (1.44–1.47)	1.31 (1.30–1.32)	1.29 (1.28–1.30)	1.25 (1.23–1.27)
Myocardial infarction						
FLI (Q1)	1471	3.27	Ref	Ref	Ref	Ref
FLI (Q2)	2949	6.58	2.01 (1.89–2.14)	1.48 (1.39–1.58)	1.47 (1.38–1.57)	1.37 (1.29–1.46)
FLI (Q3)	4917	10.99	3.36 (3.17–3.57)	2.10 (1.98–2.22)	2.08 (1.96–2.21)	1.79 (1.68–1.91)
FLI (Q4)	7237	16.24	4.97 (4.70–5.26)	2.86 (2.70–3.03)	2.78 (2.62–2.94)	2.16 (2.01–2.31)
p for trend			< 0.0001	< 0.0001	< 0.0001	< 0.0001
Per one SD increase in FLI	16,574	9.25	1.56 (1.54–1.58)	1.38 (1.36–1.40)	1.37 (1.34–1.38)	1.24 (1.22–1.27)
Stroke						
FLI (Q1)	2517	5.60	Ref	Ref	Ref	Ref
FLI (Q2)	4677	10.44	1.87 (1.78–1.96)	1.33 (1.26–1.39)	1.33 (1.26–1.39)	1.33 (1.27–1.40)
FLI (Q3)	6645	14.86	2.66 (2.54–2.78)	1.64 (1.56–1.71)	1.63 (1.56–1.71)	1.62 (1.54–1.70)
FLI (Q4)	8449	18.95	3.40 (3.25–3.55)	2.10 (2.01–2.20)	2.06 (1.96–2.15)	2.01 (1.90–2.13)
p for trend			< 0.0001	< 0.0001	< 0.0001	< 0.0001
Per one SD increase in FLI	22,288	12.45	1.39 (1.38–1.41)	1.28 (1.26–1.29)	1.26 (1.24–1.28)	1.26 (1.23–1.28)
CV mortality						
FLI (Q1)	882	1.96	Ref	Ref	Ref	Ref
FLI (Q2)	1490	3.32	1.87 (1.80–1.93)	1.35 (1.30–1.40)	1.35 (1.30–1.39)	1.31 (1.26–1.36)
FLI (Q3)	1973	4.40	2.77 (2.68–2.86)	1.71 (1.66–1.77)	1.71 (1.65–1.77)	1.60 (1.54–1.66)
FLI (Q4)	2803	6.27	3.79 (3.67–3.91)	2.27 (2.20–2.35)	2.22 (2.15–2.29)	1.98 (1.90–2.06)
p for trend			< 0.0001	< 0.0001	< 0.0001	< 0.0001
Per one SD increase in FLI	7148	3.98	1.43 (1.40–1.46)	1.24 (1.21–1.27)	1.22 (1.20–1.25)	1.28 (1.24–1.32)

Model 1: Adjusted for age and sex

Model 2: Model 1 plus current smoking, regular exercise, and income

Model 3: Model 2 plus body weight, total cholesterol, hypertension, diabetes, and use of medication for dyslipidemia

*HR* hazard ratios, *FLI* fatty liver index, *SD* standard deviation

### Subgroup analysis

**Fig. 2:**
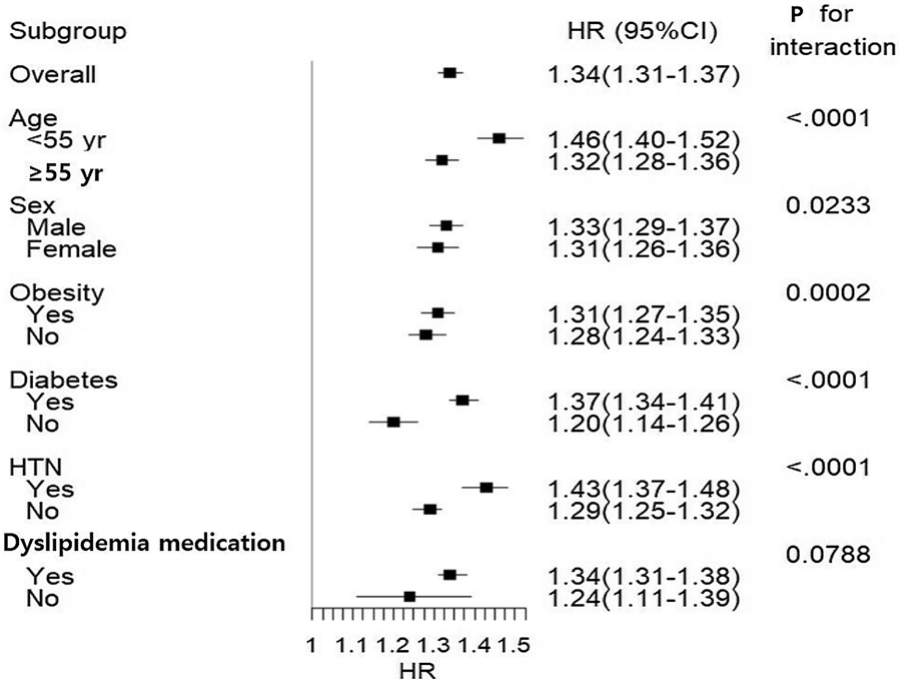
Hazard ratios and 95% confidence intervals of the primary endpoint in the highest quartile (Q4) compared to those in the lower three quartiles (Q1, Q2, and Q3) of the fatty liver index of subgroups. *Sdjusted for age, sex, current smoking, regular exercise, income, body weight, total cholesterol, hypertension, diabetes, and use of medication for dyslipidemia

Because subjects with higher FLI values were at higher risk for CVD when compared to those with lower FLI values, we further conducted analyses stratified by age, sex, obesity, diabetes mellitus, hypertension, and the use of lipid-lowering agents (Fig. 2 and Additional file 4). The highest FLI quartile group (Q4) remained predictive of newly developed non-fatal MI, stroke, and CV death in all subgroups when compared with Q1–3 groups. This finding indicated that significant associations between higher FLI and future CV events existed in all subgroups. Higher adjusted HRs of CV events were observed among those were younger (younger than 55 years), male, obese, and had diabetes and hypertension. The lipid-lowering agent subgroup did not show any significant differences in the association between the FLI and risk of CV events, except MI.

## Discussion

### Main findings of this study

In this large-scale, nationwide, longitudinal cohort study, we investigated the relationship between the FLI, a validated surrogate marker of NAFLD, and future CV events for subjects without pre-existing MI and ischemic stroke. We found that the FLI was an independent predictor of CV events, even after adjusting for possible confounding factors including body weight and cholesterol levels, during a median follow-up period of 6 years. There was a linear association between the increase in FLI values and primary outcome measures. When this association was stratified by outcome, a higher FLI value was significantly associated with an increased risk of non-fatal MI, non-fatal ischemic stroke, and CV death. We also demonstrated a greater impact of the FLI on subjects with other co-morbidities such as hypertension and diabetes. To our knowledge, the current study is the largest to date to evaluate the relationship between a clinical marker of NAFLD and future CV events in the general population.

### FLI is correlated with the CVD incidence in the general population

NAFLD is recognized as a risk factor for CVD [18]. A recent meta-analysis demonstrated that the presence of NAFLD was significantly associated with a 64% increased risk of a composite endpoint of CVD [19]. Furthermore, a cross-sectional study of 3270 subjects who were referred for coronary angiography reported that high FLI values were independently associated with increased risk of all-cause mortality, CV death, non-CV mortality, and cancer [20]. To determine the effect of NAFLD on CVD incidence in the general population, we used the FLI. The proportion of patients with newly developed CV events in our study gradually increased across FLI quartiles and FLI deciles. We also observed that a one standard deviation increase in the FLI values was associated with increased risks of CV events. Moreover, we found that linear relationship between hepatic steatosis index (HSI), other previously validated index for hepatic steatosis [21], and the CVD incidence (Additional file 5). These findings suggest a quantitative relationship, and the extent of hepatic steatosis had a major role in the development of CVD. When this association was stratified by the presence or absence of various CV risk factors (e.g., old age, obesity, diabetes, hypertension, and use of anti-dyslipidemia agents), the close relationship between higher FLI values and future risk of CVD remained. Because the NHIS database includes the entire South Korean population, our findings provide robust evidence regarding the association between the FLI and risk of CVD events in the general population, thereby suggesting that the FLI could be applied as a useful screening tool for predicting the CVD incidence in the general population.

### FLI, a surrogate marker of NAFLD, is associated with CV death

Despite the known close relationship between NAFLD and CVD [22], whether NAFLD independently increases the risk of CV death remains controversial. Several studies demonstrated unequivocally increased incidence of CV deaths among patients with NAFLD [23, 24]. Nevertheless, some meta-analyses failed to confirm this association [19, 25]. Moreover, Hwang et al. reported that the association between NAFLD and mortality caused by CVD was observed only for women [26]. Furthermore, in a 15-year follow-up study of 2075 middle-aged Caucasian subjects, the FLI was not independently associated with CVD mortality; however, it was a significant predictor of an increased risk for liver-related mortality [27]. However, previous studies involved specific cohorts with relatively small numbers of patients. Consequently, the findings of these studies have limited generalizability to a general population. Conversely, the current study was a large-scale population-based study. We demonstrated that the FLI is associated with mortality caused by CVD independent of traditional CV risk factors such as body weight, cholesterol levels, hypertension, diabetes, and use of medication for dyslipidemia. We also observed that the association between higher FLI values and CV death is significant for both sexes. It is important to determine whether NAFLD also affects future CV deaths, and our study contributes supportive and confirmative data regarding this emerging issue.

### Possible mechanisms of the independent association between FLI and CVD

Previously, NAFLD was regarded as a hepatic manifestation of metabolic syndrome, which is a traditional CVD risk factor [28, 29]. The specific contribution of NAFLD to increased CVD risk, especially in clinical studies, is difficult to assess separately from the combination of risk factors that are shared by NAFLD and CVD [30]. However, increasing evidence has suggested that NAFLD is an independent risk factor for CVD. In addition to genetic factors, various hepatokines related to the liver-gut axis and systemic insulin resistance can induce endothelial cell deterioration due to inflammatory reactions and oxidative stress, structural changes in blood vessels, and changes in blood coagulation factors [31]. Although these mechanisms plausibly link NAFLD to the development and progression of CVD, no study to date has proven a cause-and-effect relationship between these two entities. Therefore, further research is required to gain mechanistic insights regarding the pathophysiology linking NAFLD to the development and progression of these extrahepatic chronic diseases.

### Limitations

The major strengths of the current study were its large sample size, with more than 3000,000 subjects, and longitudinal data. However, several limitations of this study should be addressed. The mortality rate was assessed during a short follow-up period of 6 years, which may have been a limitation. Another limitation of our study was the use of the FLI as a surrogate measure of NAFLD instead of histological assessment of NAFLD. Furthermore, because FLI comprises known CV risk factors (BMI, triglyceride levels, waist circumference) [28, 32], these variables account for the associations observed in the current study. However, to overcome this limitation, we conducted analyses stratified by the presence or absence of these CV risk factors. Because the NHIS database relies on the assignment of a diagnostic code for CVD by physicians, there is the possibility of misdiagnoses of CVD, which may lead to under or overestimation of the disease prevalence. We did not collect data regarding medications or interventions, including weight reduction, that may have affected liver fat accumulation during the follow-up period. Moreover, other unreported confounders, including socioeconomic status and genetic factors, may have affected the association between NAFLD and mortality in our study participants. Finally, because our study subjects were mostly Korean, the results might not be generalizable to other ethnic groups.

## Conclusions

In our nationwide population-based cohort study, we observed that the FLI, a surrogate marker of NAFLD, is an independent predictor of the development of MI, ischemic stroke, and CV mortality. A linear relationship was noted between the FLI and adverse outcome measures. All relationships were independent of multiple cardio-metabolic risk factors across a wide range of patient populations. Our findings suggest that the FLI is an important predictor of major adverse CV outcomes, including CV death, in the general population. Further prospective studies are warranted to evaluate whether a quantitative relationship between NAFLD and CV events exists to determine whether early treatment of hepatic steatosis can prevent the occurrence of CVD.

## Supplementary information

**Additional file 1.** Study population

**Additional file 2.** Hazard ratios and 95% confidence intervals of cardiovascular disease mortality according to the fatty liver index quartiles, estimated by Fine-Gray regression.

**Additional file 3.** Incidence rates, hazard ratios, and 95% confidence intervals of myocardial infarction, stroke, and cardiovascular disease mortality by deciles of fatty liver index.

**Additional file 4.** Hazard ratios and 95% confidence intervals of myocardial infarction, stroke, and cardiovascular disease mortality in the highest quartile(Q4) vs. lower three quartiles of fatty liver index in subgroups.

**Additional file 5.** Risk of primary endpoints (non-fatal myocardial infarction, ischemic stroke, or cardiovascular mortality) according to baseline hepatic steatosis index (HSI) quartiles.

## Data Availability

The datasets generated and analyzed during the current study are not publicly available due to the rules of the Korean National Health Insurance System.

## Abbreviations

NAFLD: Non-alcoholic fatty liver disease
CVD: Cardiovascular disease
FLI: Fatty liver index
MACE: Major adverse cardiac events
MI: Myocardial infarction
NHIS: National Health Insurance Service
BMI: Body mass index
ANOVA: One-way analysis of variance
HR: Hazard ratios
95% CI: 95% confidence interval
