# Supplementary figures and images for: Fatty liver index and development of cardiovascular disease in Koreans without pre-existing myocardial infarction and ischemic stroke: a large population-based study
Source: Cardiovasc Diabetol. 2020 May 2;19:51. doi: 10.1186/s12933-020-01025-4 (PMC7196226; doi:10.1186/s12933-020-01025-4)

**Additional file 1. Study population**

**
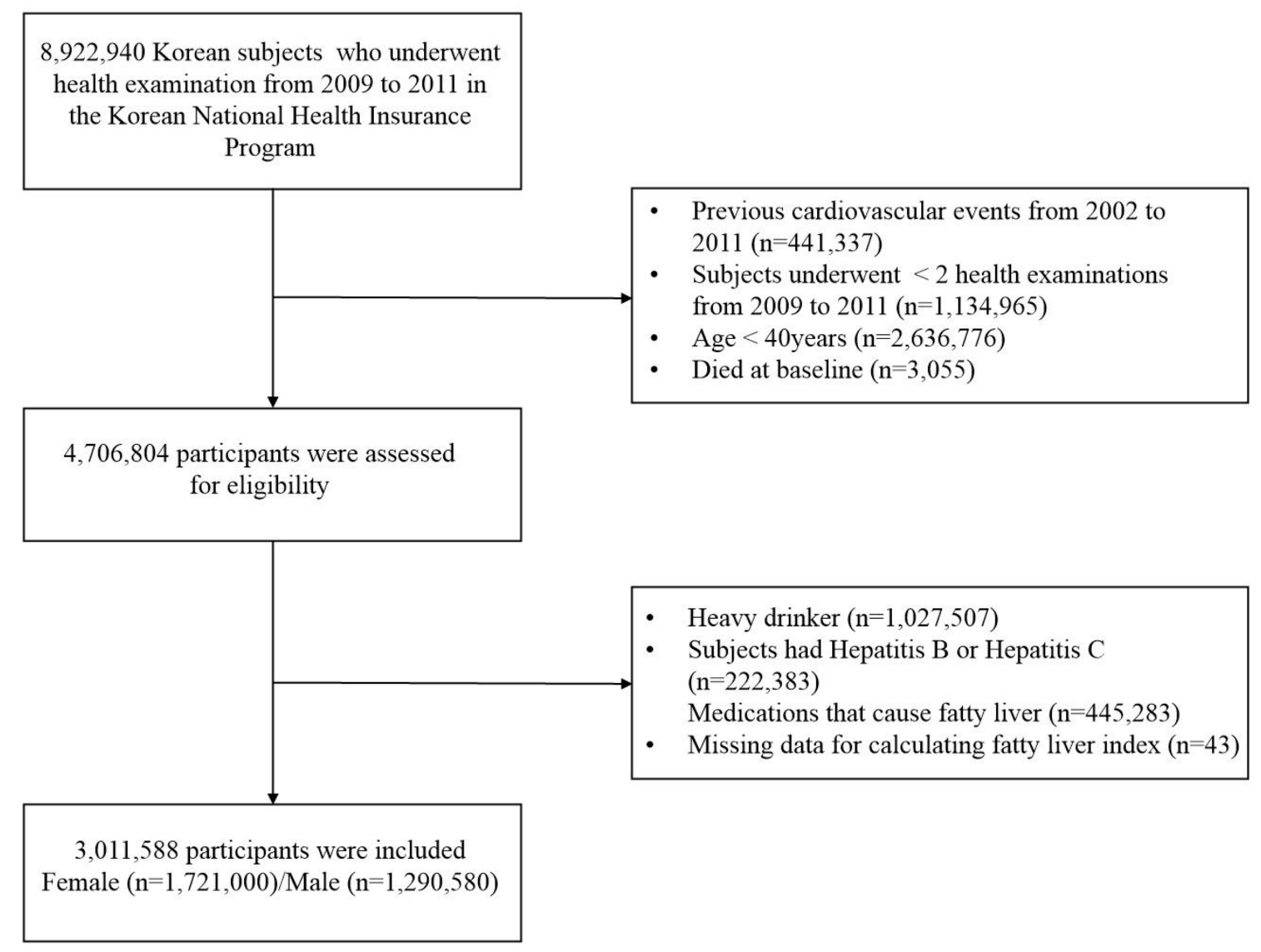
**

Supplement: Supplementary file 1 — Additional file 1. Study population [file 12933_2020_1025_MOESM1_ESM.doc]
