# Supplementary material for: Fatty liver index and development of cardiovascular disease in Koreans without pre-existing myocardial infarction and ischemic stroke: a large population-based study
Source: Cardiovasc Diabetol. 2020 May 2;19:51. doi: 10.1186/s12933-020-01025-4 (PMC7196226; doi:10.1186/s12933-020-01025-4)
Supplement: Supplementary file 2 — Additional file 2. Hazard ratios and 95% confidence intervals of cardiovascular disease mortality according to the fatty liver index quartiles, estimated by Fine-Gray regression. [file 12933_2020_1025_MOESM2_ESM.doc]

**Additional file 2. Hazard ratios and 95% confidence intervals of cardiovascular disease mortality according to the fatty liver index quartiles, estimated by Fine-Gray regression.**

|  | Unadjusted model  HR(95% CI) | Adjusted model HR(95% CI) | | |
| --- | --- | --- | --- | --- |
|  |  | Model 1 | Model 2 | Model 3 |
| FLI (Q1) | Ref | Ref | Ref | Ref |
| FLI (Q2) | 1.69(1.55-1.84) | 1.28(1.04-1.23) | 1.13(1.04-1.23) | 1.16(1.06-1.26) |
| FLI (Q3) | 2.24(2.07-2.43) | 1.23(1.13-1.33) | 1.24(1.14-1.34) | 1.26(1.15-1.38) |
| FLI (Q4) | 3.19(2.96-3.45) | 1.65(1.53-1.79) | 1.62(1.50-1.75) | 1.67(1.52-1.84) |

Model 1: Adjusted for age and sex

Model 2: Model 1 plus current smoking, regular exercise, and income

Model 3: Model 2 plus body weight, total cholesterol, hypertension, diabetes, and use of medication for dyslipidemia

HR, hazard ratios; FLI, fatty liver index; SD, standard deviation
