# Supplementary material for: Fatty liver index and development of cardiovascular disease in Koreans without pre-existing myocardial infarction and ischemic stroke: a large population-based study
Source: Cardiovasc Diabetol. 2020 May 2;19:51. doi: 10.1186/s12933-020-01025-4 (PMC7196226; doi:10.1186/s12933-020-01025-4)
Supplement: Supplementary file 3 — Additional file 3. Incidence rates, hazard ratios, and 95% confidence intervals of myocardial infarction, stroke, and cardiovascular disease mortality by deciles of fatty liver index. [file 12933_2020_1025_MOESM3_ESM.doc]

**Additional file 3. Incidence rates, hazard ratios, and 95% confidence intervals of myocardial infarction, stroke, and cardiovascular disease mortality by deciles of fatty liver index.**

**
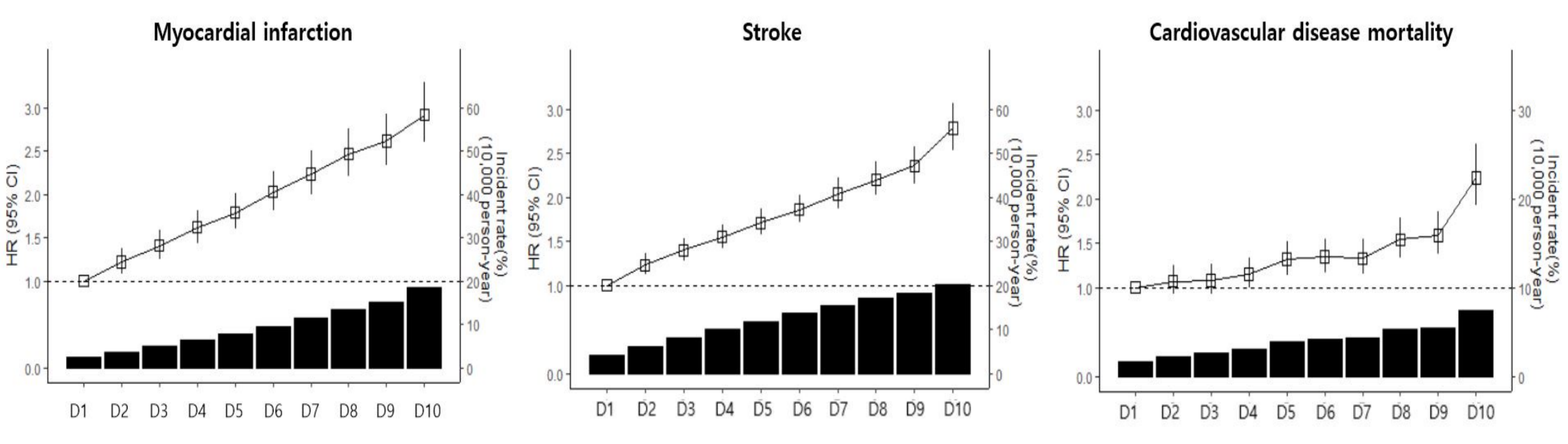
**

*adjusted for age, sex, current smoking, regular exercise, income, body weight, total cholesterol, hypertension, diabetes, and medication for dyslipidemia)
