# Supplementary material for: Fatty liver index and development of cardiovascular disease in Koreans without pre-existing myocardial infarction and ischemic stroke: a large population-based study
Source: Cardiovasc Diabetol. 2020 May 2;19:51. doi: 10.1186/s12933-020-01025-4 (PMC7196226; doi:10.1186/s12933-020-01025-4)
Supplement: Supplementary file 4 — Additional file 4. Hazard ratios and 95% confidence intervals of myocardial infarction, stroke, and cardiovascular disease mortality in the highest quartile(Q4) vs. lower three quartiles of fatty liver index in subgroups. [file 12933_2020_1025_MOESM4_ESM.doc]

**Additional file 4. Hazard ratios and 95% confidence intervals of myocardial infarction, stroke, and cardiovascular disease mortality in the highest quartile(Q4) vs. lower three quartiles of fatty liver index in subgroups.**


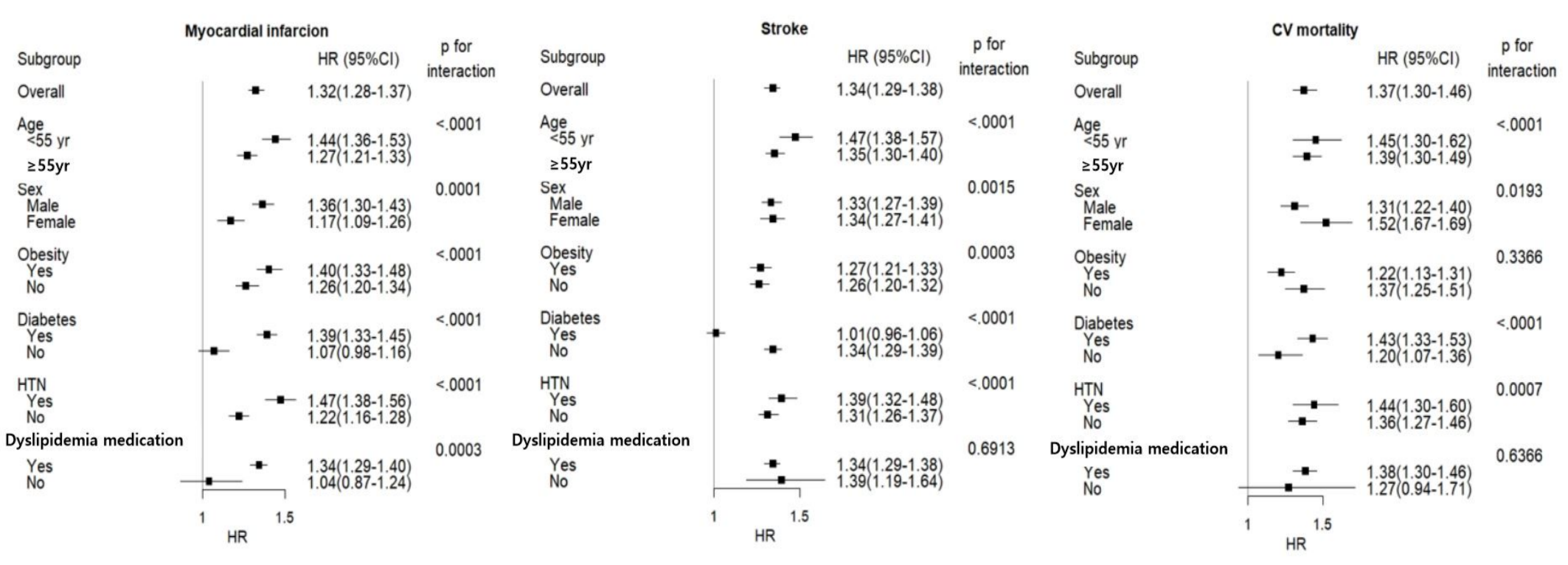


*adjusted for age, sex, current smoking, regular exercise, income, body weight, total cholesterol, hypertension, diabetes, and use of medication for dyslipidemia)

HTN, hypertension; CV, cardiovascular disease
