# Supplementary material for: Fatty liver index and development of cardiovascular disease in Koreans without pre-existing myocardial infarction and ischemic stroke: a large population-based study
Source: Cardiovasc Diabetol. 2020 May 2;19:51. doi: 10.1186/s12933-020-01025-4 (PMC7196226; doi:10.1186/s12933-020-01025-4)
Supplement: Supplementary file 5 — Additional file 5. Risk of primary endpoints (non-fatal myocardial infarction, ischemic stroke, or cardiovascular mortality) according to baseline hepatic steatosis index (HSI) quartiles. [file 12933_2020_1025_MOESM5_ESM.doc]

**Additional file 5. Risk of primary endpoints (non-fatal myocardial infarction, ischemic stroke, or cardiovascular mortality) according to baseline hepatic steatosis index (HSI) quartiles**

| Primary outcome | Unadjusted | Adjusted model HR (95% CI) | | |
| --- | --- | --- | --- | --- |
|  |  | Model 1 | Model 2 | Model 3 |
| HSI (Q1) | Ref | Ref | Ref | Ref |
| HSI (Q2) | 1.08(1.05-1.11) | 1.11(1.08-1.14) | 1.16(1.13-1.19) | 1.08(1.05-1.11) |
| HSI (Q3) | 1.30(1.26-1.33) | 1.29(1.25-1.32) | 1.35(1.32-1.39) | 1.18(1.15-1.22) |
| HSI (Q4) | 1.56(1.52-1.60) | 1.62(1.58-1.66) | 1.68(1.63-1.72) | 1.32(1.28-1.37) |
| Per one SD increase in HSI | 1.048(1.045-1.050) | 1.050(1.047-1.052) | 1.051(1.048-1.053) | 1.04(1.033-1.042) |

Model 1: Adjusted for age and sex

Model 2: Model 1 plus current smoking, regular exercise, and income

Model 3: Model 2 plus body weight, total cholesterol, hypertension, diabetes, and use of medication for dyslipidemia

HSI, hepatic steatosis; HR, hazard ratio; SD, standard deviation
